# Effect of xylitol on *Porphyromonas gingivalis*: A systematic review

**DOI:** 10.1002/cre2.724

**Published:** 2023-03-09

**Authors:** Sau You Chen, Joshua Delacruz, Yong Kim, Roger Kingston, Laura Purvis, Dileep Sharma

**Affiliations:** ^1^ Department of Periodontics, College of Medicine and Dentistry James Cook University Townsville Queensland Australia; ^2^ Discipline of Oral Health Thearpy, School of Health Sciences, College of Health, Medicine and Wellbeing The University of Newcastle Ourimbah New South Wales Australia; ^3^ Australian Institute of Tropical Health and Medicine James Cook University Townsville Queensland Australia

**Keywords:** cytokines, periodontal disease, *Porphyromonas gingivalis*, xylitol

## Abstract

**Objective:**

This review was conducted to assess the effectiveness of xylitol against *Porphyromonas gingivalis* anaerobic species, a key microbe contributing to periodontal disease pathogenesis.

**Material and Methods:**

Relevant studies published on seven online databases (Cochrane, Ovid, Pubmed, Pubmed Central, Scopus, Google Scholar, and Web of Science) were included in accordance with the PRISMA guidelines. Inclusion criteria allowed all study designs involving xylitol and P. gingivalis, literature published since the year 2000, and all xylitol delivery forms.

**Results:**

The initial search yielded 186 papers. After the removal of duplicates, five reviewers screened every article for eligibility and seven articles were selected for data extraction. Four out of seven included studies assessed the dose‐dependent effect of xylitol on *P. gingivalis* growth, two studies assessed the effect of xylitol on *P. gingivalis*‐induced cytokine expression, and one study assessed both domains.

**Conclusions:**

From the in vitro studies included in this systematic review, there is some evidence of xylitol's inhibitory effect on *P. gingivalis*. However, more evidence derived from in vivo studies is required to confirm its effectiveness warranting their routine use.

**Abbreviations**
*A. Actinomycetemcomitans*
: *Aggregatibacter actinomycetemcomitans; BANA, N‐benzoyl‐DL‐arginine‐2‐napthylamide*
CD80: cluster of differentiation 80
CD86: cluster of differentiation 86
ELISA: enzyme‐linked immunosorbent assay
GM‐0111: modified glycosaminoglycan
IL‐12: interleukin‐12
IL‐1β: interleukin‐ 1β
IL‐6: interleukin‐6
Imaris: Interactive Microscopy Image Analysis Software
LPS: lipopolysaccharides
MeSH: medical Subject Headings
MMP‐1: matrix metalloproteinase‐1
mRNA: messenger ribonucleic acid
NF‐κB: nuclear factor kappa‐light‐chain‐enhancer of activated B cells
NO: nitric oxide
OD: optical density
OTC: over‐the‐counter
PBS: Papillary Bleeding Scores
PRISMA: Preferred Reporting Items for Systematic Reviews and Meta‐Analyses
*P. gingivalis*
: *Porphyromonas gingivalis*
*S. Gordonii*
: *Streptococcus gordonii; THP‐1, Human acute monocytic leukemia cell line*
TNF‐α: tumor necrosis factor‐alpha

## 1 INTRODUCTION

Xylitol is a white crystalline sugar alcohol that occurs naturally in a variety of plant species including plum, strawberry, and pumpkin (Nayak et al., 2014). The compound initially gained popularity as a sugar substitute during World War II, when a shortage of sucrose (table sugar) was being experienced in Finland (Rumbeiha & Snider, 2014). Throughout the 1960s, its use became more widespread across Europe and it was approved for use by the United States Food and Drug Administration in 1963 (Nayak et al., 2014). The sugar alcohol xylitol has been steadily growing in use as an artificial sweetener ever since. However, the relationship between xylitol and caries was discovered with the first major study examining the effect of xylitol on cariogenic bacteria, that is, the Turku Sugar study published in 1974 (Scheinin et al., 1974). The Turku study assessed the effects of sucrose, fructose, and xylitol on the incidence of caries in human dentition. It was revealed that study participants who substituted xylitol for sucrose experienced a drastic reduction in the development of dental caries over a 2‐year period (Scheinin et al., 1974). Many studies have since been published, confirming and further exploring the effect of xylitol on oral microbiota (Nayak et al., 2014; Rafeek et al., 2019; Söderling, 2009; Wu et al., 2022). Perhaps the most well‐known health benefit is its effect on *Streptococcus mutans*, a facultative anaerobe considered to be the main bacterial species responsible for dental caries. Streptococci species such as *S. Mutans* are known to transport xylitol into the cell via the fructose phosphotransferase system (Na et al., 2013). Xylitol is subsequently expelled from the bacterial cell with a net loss of energy from xylitol metabolism, resulting in bacterial death (Nayak et al., 2014).

Periodontitis is a chronic inflammatory disease characterized by recurrent infection and inflammation that involves alveolar bone loss (Savage et al., 2016). Periodontitis is a result of host immune responses triggered by endotoxins released from bacterial plaque (Han et al., 2005). A healthy periodontium maintains local tissue homeostasis by balancing its immune response to the local microbial ecosystem (Savage et al., 2016). Dysbiotic microbial ecosystems can result if the homeostasis is disrupted by changes to the microbial environment within the dental biofilm (Savage et al., 2016). The immune response is then heightened, causing more tissue destruction and exacerbating the proliferation of pathogenic microorganisms (Savage et al., 2016). Extensive research in this area has identified microorganisms such as *Porphyromonas gingivalis* and *Aggregattibacter actinomycetemcomitans* as major contributors to the pathogenesis of periodontitis (Åberg et al., 2015; Casarin et al., 2010; Jain & Darveau, 2010; Savage et al., 2016; Schacher et al., 2007; Xu et al., 2020). These pathogenic bacteria are known to be the cause of sustained inflammation, which enhances osteoclastic activity, resulting in periodontal bone loss (Savage et al., 2016). Although there have been a significant number of studies assessing the effects of xylitol on cariogenic bacteria, minimal research has been conducted to assess its potential effect on periodontal disease.

*P. gingivalis* is a Gram‐negative anaerobic bacterium that has been reported to play a major role in the initiation and progression of periodontitis (Han et al., 2005). It causes destruction of periodontal tissues through a combination of proteinases and heightened host response to these bacterial products (Han et al., 2005). In response to infection, immune cells such as monocytes and neutrophils migrate to the periodontium to produce cytokines and chemokines that activate the adaptive immune response (Na et al., 2018). Neutrophils, the main leukocyte in the gingival crevice, prevent bacteria from invading the underlying tissue (Na et al., 2018). However, they have been suggested to induce excessive immune responses in chronic pathological conditions such as periodontitis, which may induce excessive tissue destruction through the overproduction of cytokines (Na et al., 2018). Therefore, studies assessing how xylitol impacts the production of cytokines from various immune cells stimulated by *P. gingivalis* provide insight into its role in reducing periodontitis. The focus research question for this systemic review was “Does Xylitol suppress the growth of *P. gingivalis*?” A secondary question included “Does Xylitol influence inflammatory response induced by *P. gingivalis*?” In addition, the objective is to evaluate the effect of xylitol on *P. gingivalis* and determine its relevance in managing periodontal disease through direct action on *P. gingivalis* or on *P. gingivalis*‐induced cytokine expression.

## 2 MATERIALS AND METHODS

This systematic review was conducted following the Preferred Reporting Items of Systematic Reviews and Meta‐Analyses (PRISMA 2020) guideline to address the proposed research questions (Page et al., 2021). This systematic review was registered on PROSPERO (ID‐CRD42022348877) and can be assessed on: https://www.crd.york.ac.uk/prospero/display_record.php?ID=CRD42022348877.

### 2.1 Information sources and search strategy

The following databases were used to perform a comprehensive literature search: Web of Science, Scopus, Ovid, Pubmed, PubMed Central, Cochrane, and Google Scholar, with the last search conducted in July 2022. The MeSH terms used to retrieve the articles are listed in Table 1. All records were exported into EndNote v20 and after removal of duplicates, articles were excluded based on the inclusion and exclusion criteria.

**Table 1:** MeSH terms used to search databases.

Database	MeSH
Web of science	“porphyromonas gingivalis” OR “p. gingivalis” OR “p gingivalis” (Topic) and xylitol OR “birch sugar” (Topic)
Scopus	(TITLE‐ABS‐KEY (xylitol) OR TITLE‐ABS‐KEY (“birch sugar”) AND TITLE‐ABS‐KEY (porphyromonas AND gingivalis) OR TITLE‐ABS‐KEY (p. AND gingivalis) OR TITLE‐ABS‐KEY (p AND gingivalis))
PubMed	(xylitol[Title/Abstract] OR “birch sugar”[Title/Abstract]) AND (“porphyromonas gingivalis”[Title/Abstract] OR “p. gingivalis”[Title/Abstract] OR “p gingivalis”[Title/Abstract])
PMC	((“birch sugar”[Body ‐ All Words] OR xylitol[Body ‐ All Words])) AND (“porphyromonas gingivalis”[Body ‐ All Words] OR “P. gingivalis”[Body ‐ All Words] OR “p gingivalis”[Body ‐ All Words])
Ovid	1. exp porphyromonas gingivalis 2. exp Xylitol 3. 1 and 2
Cochrane	(xylitol OR “birch sugar”) AND (“p. gingivalis” OR “porphyromonas gingivalis” OR “p gingivalis”) in Title Abstract Keyword
Google scholar	xylitol OR “birch sugar” AND “porphyromonas gingivalis” OR “p. gingivalis” OR “p gingivalis”

### 2.2 Eligibility criteria

Studies published between January 1, 2000, and July 11, 2022, were considered for inclusion. To capture the current evidence on the relationship between xylitol and *P. gingivalis*, wide‐ranging inclusion criteria were established. Study designs including randomized‐controlled studies and controlled clinical studies were included. The inclusion also required the publication of the study in English and did not require ethics approval to be included, so long as it was published between January 1, 2000, and July 11, 2022. All xylitol delivery forms were considered for inclusion. Exclusion criteria were studies not published in the English language, nonpeer‐reviewed studies, case reports, reviews of any type, studies wherein xylitol was combined with other agents, and studies that did not assess *P. gingivalis* exclusively.

### 2.3 Study selection process

**Figure 1:**
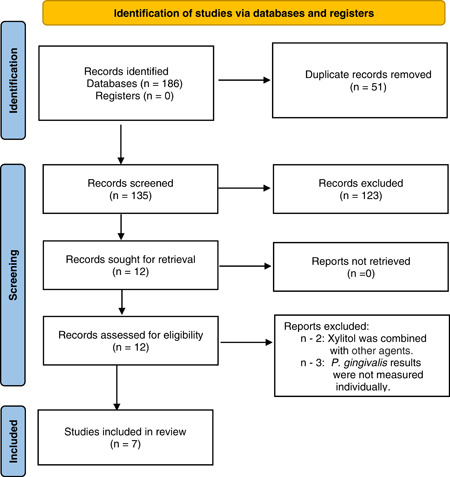
Flow diagram of the study selection process according to the Preferred Reporting Items for Systematic Reviews and Meta‐Analyses guideline (Page et al., 2021).

The selection process utilized in this systematic review has been depicted in a PRISMA flow diagram (Figure 1). The same MeSH terms were used to search each database (Table 1). The initial search yielded 186 studies in total. After removing 51 duplicates, the titles and abstracts of the remaining articles were screened by all reviewers (L. P., J. D., S. Y. C., R. K., Y. K.). Following this, 12 articles remained, and their full text was assessed for eligibility by all reviewers. Any disagreement was discussed with the other author (D. S.) and the decision of inclusion or exclusion was made. A total of seven articles fulfilled all eligibility criteria and were included in the systematic review.

### 2.4 Data collection process

The information collected from the selected studies was compiled into a custom‐designed spreadsheet (see Table 2). The results included study, objective, sample, intervention/xylitol treatment concentration, method of treatment administration, parameter/time assessed, and the outcome of the experiment. The collected data were reviewed by all reviewers (L. P., J. D., S. Y. C., R. K., Y. K.). Any differences between reviewers were resolved by further discussions with the senior author (D. S.) to arrive at a consensus.

**Table 2:** Characteristics of in vitro studies included in the systematic review.

Study	Objective	Sample	Intervention	Methodology	Parameter	Outcome
Han et al. (2005)	To assess the effects of xylitol on the growth of *Porphyromonas gingivalis*‐ and LPS‐induced cytokine expression	Mouse macrophage cells (RAW 264.7 cells) treated with *P. gingivalis* LPS	1, 2, 4, or 8% xylitol for *P. gingivali*s LPS‐induced cytokine expression 0, 2.5, 5, 10, or 20% xylitol for growth of *P. gingivalis*	Cytokine expression	TNF‐α assessed after 1 h IL‐1β assessed after 4 h NF‐κB assessed after 30 min *P. gingivalis* growth assessed after 24 h	4% and 8% xylitol inhibited TNF‐α and IL‐1β expression NF‐κB activation inhibited by xylitol in a dose‐dependent manner Growth of *P. gingivalis* inhibited in a dose‐dependent manner and entirely blocked by 20% xylitol
				○ RAW 264.7 cells pretreated with xylitol 30 min before treatment with 1 g of LPS/mL and measured using the ELISA kit.		
				Growth		
				○ *P. gingivalis* cultured for 24 h with xylitol and measured using a spectrophotometer		
Park et al. (2014)	To investigate the anti‐inflammatory effects of xylitol in periodontal infections	Human cell line THP‐1‐derived macrophages pretreated with *P. gingivalis*	3% xylitol	Immunosorbent assay and multiplex assay kit measured the production of cytokines Phagocytosis and NO production measured using the phagocytosis assay, viable cell count, and Griess reagent *P. gingivalis* adhesion determined by immunostaining Costimulatory molecule expression examined by flow cytometry	Cytokines were assessed after 6 or 24 h Cell adhesion and internalization of *P. gingivalis* measured after 90 min or 6 h Phagocytic function measured after 2 h CD80 and CD86 molecules measured after 90 min	Release of TNF‐α and IL‐1β inhibited significantly by 3% xylitol Xylitol inhibited the production of inflammatory cytokines, chemokines, and NO Adhesion and internalization of *P. gingivalis* to macrophages significantly inhibited by xylitol Xylitol inhibited phagocytic function against *P*. *gingivalis* Xylitol did not alter upregulation of CD80 and CD86 expression
Na et al. (2018)	To investigate the anti‐inflammatory effects of xylitol against *P. gingivalis* infection in neutrophils	Mouse neutrophils pretreated with *P. gingivalis*	1%, 2%, 5% xylitol	Neutrophils pretreated with xylitol 30 min before treatment with *P. gingivalis*. ELISA kit used to analyze cytokine levels	IL‐1β, IL‐6, and TNF‐α assessed after 24 h	Xylitol significantly suppressed the production of IL‐1β, IL‐6, and TNF‐α
Na et al. (2013)	To evaluate the growth of *P. gingivalis* among other oral bacteria when xylitol was added	*P. gingivalis*	5%, 10%, or 15% xylitol	*P. gingivalis* growth measured via OD using the ELISA reader	*P. gingivalis* incubated for 1 to 4 days, with OD measurements taken during this time	5%, 10%, and 15% xylitol showed mild, marked, and complete growth inhibition, respectively
Badet et al. (2008)	To examine the effect of xylitol on biofilm bacterial species (including *P. gingivalis*)	*P. gingivalis*	1%, 3% xylitol added to the sample before anaerobic incubation	Hydroxyapatite discs processed using confocal laser scanning microscopy and a sterile instrument. Harvested biofilms were diluted and plated	*P. gingivalis* colony‐forming units counted after 48h incubation	*P. gingivalis* not recovered in either concentrations
Savage et al. (2016)	To investigate whether GM‐0111 disrupts the growth and biofilm formation of *P. gingivalis* and *Aggregatibacter actinomycetemcomitans* with hyaluronic acid or xylitol	*P. gingivalis*	Treatment groups tested with GM‐0111 molecules with hyaluronic acid or xylitol at 1%, 2%, 4%, and 8% w/v concentrations	Bacteria directly counted using the Guava HT‐8 flow cytometer	*P. gingivalis* samples sonicated for 5 min in an ultrasonic bath before counting	Authors reported “negligible effect” of xylitol on *P. gingivalis* growth.
Hashino et al. (2013)	To examine the effects of sugar alcohols (including xylitol) on *Streptococcus gordonii*–*P. gingivalis* mixed species biofilm development	*P. gingivalis* and *S. gordonii*–dual‐species biofilm	10% erythritol, xylitol, or sorbitol	Analysis of biofilm formation performed using confocal laser scanning microscopy. *P. gingivalis* biovolume quantified using the Imaris system	*P. gingivalis* biovolume and density measured after 24 h	Biovolume and density of *P. gingivalis* decreased

Abbreviations: CD80, cluster differentiation of 80; ELISA, enzyme‐linked immunoassay; GM‐0111, modified glycosaminoglycan; IL‐1β, interleukin‐1β; Imaris, Interactive Microscopy Image Analysis Software; LPS, lipopolysaccharides; NF‐κB, nuclear factor kappa‐light‐chain‐enhancer of activated B cells; NO, nitric oxide; OD, optical density; TNF‐α, tumor necrosis factor‐alpha

### 2.5 Quality assessment of selected studies

The quality and risk of bias of the seven studies were determined by all reviewers (L. P., J. D., S. Y. C., R. K., Y. K.) following the Modified Consort checklist shown in Table 3 (Roberts et al., 2020).

**Table 3:** Modified CONSORT checklist of items for reporting in vitro studies of dental materials (Roberts et al., 2020).

Section	Item	Domain
Abstract	1	Structured summary of trial design, methods, results, and conclusions
Introduction	2a	Scientific background and explanation of rationale
	2b	Specific objectives and/or hypotheses
Methods	3	Intervention: the intervention for each group, including how and when it was administered, with sufficient detail to enable replication
	4	Outcomes: completely defined, pre‐specified primary and secondary measures of outcome, including how and when they were assessed
	5	Sample size: how sample size was determined
	6	Randomization: method used to generate the random allocation sequence
	7	Allocation: mechanism used to implement the random allocation sequence, describing any steps taken to conceal the sequence until an intervention was assigned
	8	Implementation: who generated the random allocation sequence, (who selected teeth, and assigned teeth to intervention)
	9	Blinding: if done, who was blinded after assignment to intervention and how
	10	Statistics: statistical methods used to compare groups for primary and secondary outcomes
Results	11	For each primary and secondary outcome, results for each group, and the estimated size of the effect and its precision
Discussion	12	Trial limitations, addressing sources of potential bias, imprecision, and, if relevant, multiplicity of analyses
Other information	13	Sources of funding and other support role of funders
	14	Where the full trial protocol can be accessed, if available

## 3 RESULTS

### 3.1 Study selection

Five reviewers (L. P., J. D., S. Y. C., R. K., Y. K.) independently carried out the database searches using MeSH terms shown in Table 1. After removing duplicates, reviewers conducted screening of the abstracts and titles to identify articles that potentially fulfilled the inclusion criteria. The remaining studies were then assessed by the same five reviewers by reading the full‐text versions. Each article was assessed by two reviewers and any disagreements were resolved by having a third reviewer read the full text to avoid inter‐reviewer variability. Cohen's *κ* calculator was used to document and measure inter‐assessor agreement during the selection process. The *κ* value calculated was 0.833, meaning that the inter‐assessor agreement and reliability was strong (McHugh, 2012).

### 3.2 Study characteristics

The data extracted from the seven studies are shown in Table 2. All seven studies were in vitro studies, wherein four articles specifically tested the effect of xylitol on *P. gingivalis* growth (Badet et al., 2008; Na et al., 2013; Hashino et al., 2013; Savage et al., 2016), two articles studied the effect on the inflammatory response (Na et al., 2018; Park et al., 2014), and only one article studied its effect on both (Han et al., 2005).

### 3.3 Quality assessment of included studies

The risk of bias of the seven in vitro studies was assessed using a Modified CONSORT checklist (see Table 3) (Roberts et al., 2020). Five reviewers (J. D., L. P., R. K., S. Y. C., Y. K.) independently reviewed each of the articles and disagreements were resolved by further discussion; the final findings are presented in Table 4.

**Table 4:** Quality assessment of in vitro studies according to items of the modified CONSORT checklist (Roberts et al., 2020).

Citation	1	2a, 2b	3	4	5	6	7	8	9	10	11	12	13	14
Na et al. (2018)	Y	Y, N	Y	Y	N	N/A	N	N	N	Y	N	N	Y	N/A
Na et al. (2013)	Y	Y, Y	Y	Y	N	N/A	N	N	N	N	N	N	Y	N/A
Badet et al. (2008)	Y	Y, N	Y	Y	N	N/A	N/A	N/A	N	Y	N	N	N	N/A
Savage et al. (2016)	Y	Y, N	Y	Y	N	N	N	N	N	Y	N	N	Y	N/A
Hashino et al. (2013)	Y	Y, N	Y	Y	N	Y	N	N	N	Y	N	N	Y	N/A
Park et al. (2014)	Y	Y, N	Y	Y	N	N	N	N	N	Y	N	N	Y	N
Han et al. (2005)	Y	Y, N	Y	Y	N	N/A	N/A	N/A	N	N	N	N	Y	N/A

Abbreviations: N, failed to fulfill the criteria on the checklist, N/A, not applicable; Y, successfully fulfilled the criteria on the checklist.

### 3.4 Effect of xylitol on *P. gingivalis* growth

Five of the included articles examined the inhibitory effect of xylitol on *P. gingivalis* growth in vitro (Badet et al., 2008; Han et al., 2005; Hashino et al., 2013; Na et al., 2013; Savage et al., 2016). However, variations existed between xylitol concentrations, xylitol treatment durations, and tested specimens in these studies. Hashino et al. reported a significant reduction of *P. gingivalis* biovolume and density on a heterotypic biofilm of stained *P. gingivalis* and *Streptococcus gordonii* after suspension in a 10% w/v xylitol solution for 24 h (Hashino et al., 2013). On the contrary, Savage et al. subcultured aliquots of *P. gingivalis* and *A. actinomycetecomitans* in 1%, 2%, 4%, and 8% w/v of xylitol solution in microplate wells over an unreported period of time (Savage et al., 2016). Xylitol exerted negligible effect on *P. gingivalis* growth, although the results indicated a reduction in the *P. gingivalis* population as the xylitol concentration increased (Savage et al., 2016). Additionally, Badet et al. reported inconclusive findings from their study since no recovery of *P. gingivalis* was noted (Badet et al., 2008). Han et al. (2005) reported that the effectiveness of xylitol greatly depended on the concentration administered. This study tested *P. gingivalis* with xylitol in two separate experiments: the effect on *P. gingivalis* on lipopolysaccharides (LPS)‐induced tumor necrosis factor‐alpha (TNF‐α) and interleukin (IL)‐1β and nuclear factor kappa‐light‐chain‐enhancer of activated B cells (NF‐κB) levels as well as the direct effect on *P*. *gingivalis* growth. The concentrations of xylitol differed between the two studied outcomes. In the cytokine expression experiment, xylitol was used as 1%, 2%, 4%, and 8% w/v solutions (Han et al., 2005). In the *P. gingivalis* growth experiment, concentrations were 2.5%, 5%, 10%, and 20% w/v. It was reported that *P. gingivalis* growth was entirely inhibited in the 20% w/v solution. Weaker solutions such as the 5% and 10% w/v concentrations still showed a significant reduction in *P. gingivalis* growth of 50% and 80%, respectively (Han et al., 2005).

A similar result was reported by Na et al. (2013), who assessed *P. gingivalis* growth at 5%, 10%, and 15% w/v concentrations. All tested concentrations demonstrated a reduction in bacterial growth. 5% w/v xylitol showed mild inhibition of growth (<50% *P. gingivalis* inhibited), 10% w/v showed marked growth inhibition, and 15% showed complete bacterial inhibition. However, these samples were treated for a period extending up to 96 h, which is significantly longer than the exposure times of the other studies (24–48 h).

### 3.5 Effect of xylitol on *P. gingivalis* cytokine expression

Three of the included studies investigated the effect of xylitol on *P. gingivalis*‐induced TNF‐α and IL‐1β expression (Han et al., 2005; Na et al., 2018; Park et al., 2014). All three studies showed significant dose‐dependent inhibition of the aforementioned cytokines. One study reported that IL‐1β expression was reduced more in a 1% xylitol concentration than a 2% concentration; however, the greatest reduction in both TNF‐α and IL‐1β was demonstrated at 5%, the highest concentration used (Na et al., 2018). All three studies varied in terms of the xylitol concentration used (see Table 2). In a second study, Han et al. found that 4% and 8% xylitol concentrations substantially inhibited TNF‐α and IL‐1β mRNA expression, with only a minor reduction in 1% and 2% xylitol concentrations compared to the control group (Han et al., 2005). Specifically, 8% xylitol almost completely inhibited both TNF‐α and IL‐1β mRNA expression (Han et al., 2005). Finally, Park et al. showed that 3% xylitol was more effective in reducing TNF‐α and IL‐1β compared with lower concentrations (Park et al., 2014). The reduction was also time‐dependent as the highest level of cytokine inhibition was observed when macrophages were incubated in xylitol for 24 h compared to 6 h (Park et al., 2014). These three studies also reported that xylitol pretreatment decreased the production of other pro‐inflammatory cytokines including IL‐12, p40, eotaxin, interferon γ‐induced protein 10, monocyte chemotactic protein‐1, macrophage inflammatory protein‐1, nitrous oxide, IL‐6, and NF‐κB (Han et al., 2005; Na et al., 2018; Park et al., 2014).

In the study carried out by Park et al., *P. gingivalis* was reported to attach to the surface of THP‐1‐dervied macrophages and was internalized into cells (Park et al., 2014). Pretreatment of 3% xylitol inhibited the adhesion and internalization. It also inhibited the phagocytic function of macrophages against live *P. gingivalis*. However, the upregulated CD80 and CD86 costimulatory molecule expression induced by *P. gingivalis* was not altered by xylitol.

## 4 DISCUSSION

Previous research and multiple systematic reviews have been conducted on xylitol's role as an anticariogenic agent as it has been proven to be an effective preventive means of reducing *S. mutans* (Janakiram et al., 2017; Riley et al., 2015). In a 2017 systematic review, it was noted that xylitol was an effective preventive agent for caries development when used as an alternative over‐the‐counter (OTC) sweetener (Janakiram et al., 2017). However, there have been no systematic reviews consolidating the information on how xylitol may be beneficial in maintaining periodontal health. This is the first systematic review conducted assessing the effect of xylitol on *P. gingivalis*, a major contributing bacterial species in periodontal disease pathogenesis. The included studies investigated the anti‐periodontitis effect of xylitol, through its influence on *P. gingivalis* growth and inhibitory effect on inflammatory cytokines. Although more research is needed on this topic, five of the seven reviewed articles showed promising signs of *P. gingivalis* growth inhibition as well as the inhibition of inflammatory cytokine production due to xylitol exposure.

The two main inflammatory cytokines measured across all three studies were TNF‐α and IL‐1β, both of which are inflammatory cytokines found in inflamed periodontal tissues and are principal inducers of effector molecules that cause bone remodeling (Papathanasiou et al., 2020). They are also important markers for periodontitis progression and severity (Gomes et al., 2016). IL‐1β increases bone resorption by increasing osteoclastogenesis and exacerbates vasodilation, inflammatory cell chemotaxis, and collagen degradation (Papathanasiou et al., 2020). This is achieved through the upregulation of matrix metalloproteinase secretion (Papathanasiou et al., 2020). TNF‐α is crucial to the initiation and perpetuation of inflammatory and tissue‐destructive responses in periodontitis (Tervahartiala et al., 2001). It has the ability to activate a large number of cytokines, chemokines, cell adhesion molecules, and transcription factors (Tervahartiala et al., 2001). Other effects of TNF‐α include stimulation of the expression of matrix metalloproteinase‐1 (MMP‐1), collagenase‐2, and collagenase‐3, which degrade the periodontal ligament (Tervahartiala et al., 2001). In addition, TNF‐α and IL‐1β stimulate NO expression, which enhances the progression of periodontitis (Tervahartiala et al., 2001). The overproduction of NO is associated with periodontal pathogenic effects including direct cellular cytotoxicity and various inflammatory processes.

The seven studies in this review reported variable results supported by inconsistent levels of evidence. Three studies reported that the *P. gingivalis* population was suppressed at higher concentrations of xylitol treatments (Na et al., 2013; Han et al., 2005; Hashino et al., 2013), while one study reported negligible effect and another one was inconclusive (Badet et al., 2008; Savage et al., 2016). All three studies that assessed cytokine production demonstrated that xylitol significantly suppressed *P. gingivalis*‐induced cytokine production (TNF‐α and IL‐1β) (Han et al., 2005; Na et al., 2018; Park et al., 2014), thus supporting the role of xylitol as an anti‐inflammatory agent.

Some of the variations in results seen among the included studies may be attributed to the differences in the experimental design, preparation, cell types, and changes in exposure parameters. Although most of the included studies revealed a dose‐dependent reduction of *P. gingivalis* growth when treated with xylitol, a wide variation between the concentrations of xylitol and the degree of *P. gingivalis* suppression was evident.

The studies varied in terms of the time points at which *P. gingivalis* parameters were measured, which makes generalization of results more difficult. In the Han et al. study, the time points chosen to quantify TNF‐α and IL‐1β expression were set by performing prior tests to determine when these cytokines were at the maximum levels after mouse cells were treated with *P. gingivalis* LPS (Han et al., 2005). The time point at which the cytokines were at the maximum level was the time point at which they were assessed after treatment with xylitol. In the studies carried out by Park et al. (2014) and Na et al. (2018), the rationale for the selected time points for measuring inflammatory cytokine expression was not specified. Time points varied from 1 to 24 h across the three studies, with all showing reductions in inflammatory cytokine expression at their respective time points (Han et al., 2005; Na et al., 2018; Park et al., 2014). Additionally, none of the studies investigating *P. gingivalis* growth inhibition specified the rationale for the selected time points, which varied from 24 to 96 h across the five studies. In a clinical situation, it is unlikely that a patient would be exposed to xylitol for this length of time. It is worth considering that xylitol is generally used in chewing gum and hard candies, which would be unlikely to persist in the oral cavity for more than a few minutes to an hour (Rajapaksha et al., 2019). The effect of xylitol on *P. gingivalis* growth in a shorter period of time is yet to be determined.

All growth studies utilized variable concentration(s) of xylitol (1% to 20% w/v) (Badet et al., 2008; Han et al., 2005; Hashino et al., 2013; Na et al., 2013; Savage et al., 2016), and thus a valid comparison to find interstudy agreement or disagreement may not be possible due to these variations. From the data presented in the included studies, xylitol started to exert growth inhibition of *P. gingivalis* at 5% concentration and became more effective with increasing concentration, reaching complete inhibition at 20% concentration (Badet et al., 2008; Han et al., 2005; Hashino et al., 2013; Na et al., 2013; Savage et al., 2016). However, these observations were based on the available data and could have been influenced by other inter‐ and inter‐study variables, such as the duration of xylitol exposure. The precise (effective) concentration of xylitol needs to be deduced, based on further controlled and valid studies. Furthermore, xylitol concentration and the vehicle used vary considerably between the commercially available products and the intervention used in the studies. In addition, chewing gum and lozenges contain xylitol in concentrations ranging from 66% to 96% (turska‐szybka et al., 2016). Therefore, it is difficult to evaluate if the results from these in vitro studies reflect real‐life effectiveness of commercial products in inhibiting *P. gingivalis*.

In the Han et al. (2005) and Park et al. (2014) study, the cytotoxic effects of xylitol in different concentrations were determined by measuring cell viability. In the Han et al. study, it was reported that there was no cytolytic effect of xylitol on mouse macrophage cells (Han et al., 2005). They did not include quantitative data supporting this and also did not specify the concentration of xylitol utilized. In the Park et al. study, the survival rate of human monocytes reduced with the use of xylitol concentrations higher than 6% (Park et al., 2014). This difference may be associated with the different types of cells assessed and the concentrations of xylitol.

It is evident from the Modified CONSORT checklist that the studies included in this systematic review had a moderate to high risk of bias. It is well‐acknowledged that sample sizes that are too small can prevent the extrapolation of findings (Faber & Fonseca, 2014). Notably, one of the limitations evident in all the included studies was the lack of sample size calculation. It is important to note that some authors were involved in multiple studies included in this review. Also, the majority of authors in Savage et al. were affiliated with GlycoMira Therapeutics, Inc.™ and were testing their products in comparison to xylitol (Savage et al., 2016). However, this was more of an observation rather than a limitation.

The results obtained from the selected articles in this systematic review should be interpreted with caution since no included studies mention limitations such as potential bias and imprecision (Ross & Zaidi, 2019). Additionally, two of the studies (Han et al., 2005; Na et al., 2013) were missing statistical analysis, which undermined the validity of their results. Furthermore, six studies did not incorporate randomization into their study designs (Badet et al., 2008; Han et al., 2005; Na et al., 2013, 2018; Park et al., 2014; Savage et al., 2016). These studies did not carry out blinding before examination, which further reduced the validity of the results. There was also a questionable level of reliability as only one study reported the number (replicates) of experiments that was conducted (Han et al., 2005).

Notably, all of the articles assessed in this review involved in vitro studies, which are unable to simulate the human oral cavity and subsequently limit the clinical breadth of the studies. Although in vitro studies are crucial to assessing and measuring the specific effect of xylitol on *P. gingivalis* in a controlled environment, the inclusion of in vivo studies and human clinical trials studies would provide valuable insight on xylitol's affects on periodontal disease progression in an oral environment. Thus, it is necessary to interpret data obtained from in vitro studies with discretion. Recently, an in vivo study by Bretz et al. (2006) reported on a randomized double‐masked placebo‐controlled trial investigating the effects of different treatments (one being xylitol chewing gum) on periodontal bacteria and clinical parameters in children and mothers. They performed chairside BANA tests to detect the presence of *Treponema denticola*, *P. gingivalis*, and *Tannerella forsythia* in plaque samples. Results showed that all treatment interventions, including the use of xylitol chewing gum, yielded lower BANA scores compared to the control groups; however, no statistically significant effect was noted. It is worth noting that this study could not be included in the systematic review as data for *P. gingivalis* was not recorded individually (only the BANA score). Bretz et al. also noted that papillary bleeding scores (PBS) measured by stimulating the interdental papilla with a triangular wooden wedge were lower for all patients following treatment; however, the differences were not statistically significant.

## 5 CONCLUSION

This systematic review reported evidence supporting the inhibitory effect of xylitol on *P. gingivalis* growth and pro‐inflammatory cytokine expression, which are known contributors in the pathogenesis of periodontal disease. The seven included studies varied in experimental design, with two of the seven included studies recording negligible and inconclusive results. However, the remaining five studies drew supportive arguments for the inhibitory effect of xylitol against *P. gingivalis* growth and pro‐inflammatory cytokine expression. To confirm the clinical effectiveness of xylitol in the prevention and management of periodontal disease, further research including long‐term in vivo studies is recommended.

## AUTHOR CONTRIBUTIONS

All authors contributed equally to the review process and preparation of the manuscript.

## CONFLICT OF INTEREST STATEMENT

The authors declare no conflict of interest.

## Data Availability

The data that support the findings of this study are available from the corresponding author upon reasonable request.
